# Effects of neuromuscular electrical stimulation on voluntary muscle activation and peripheral muscle contractility following short‐term bed rest

**DOI:** 10.1113/EP092194

**Published:** 2025-03-31

**Authors:** Sofie K. Hansen, Pernille Hansen, Tania W. Berry, Hans D. Grønbæk, Camilla M. Olsen, Youssif Merhi, Shweta Agarwala, Per Aagaard, Lars G. Hvid, Jakob Agergaard, Flemming Dela, Charlotte Suetta

**Affiliations:** ^1^ Geriatric Research Unit Copenhagen University Hospital – Bispebjerg and Frederiksberg Copenhagen Denmark; ^2^ CopenAge, Copenhagen Center for Clinical Age Research University of Copenhagen Copenhagen Denmark; ^3^ Deptarment of Electrical and Computer Engineering Aarhus University Aarhus Denmark; ^4^ Department of Sport and Clinical Biomechanics Muscle Physiology and Biomechanics Research Unit, University of Southern Denmark Odense Denmark; ^5^ Exercise Biology, Department of Public Health Aarhus University Aarhus Denmark; ^6^ The Danish MS Hospitals, Ry and Haslev Copenhagen Denmark; ^7^ Institute of Sports Medicine Copenhagen, Department of Orthopedic Surgery Copenhagen University Hospital – Bispebjerg and Frederiksberg Copenhagen Denmark; ^8^ Center for Healthy Aging, Department of Clinical Medicine University of Copenhagen Copenhagen Denmark; ^9^ Xlab, Department of Biomedical Sciences University of Copenhagen Copenhagen Denmark; ^10^ Department of Physiology and Biochemistry Riga Stradins University Riga Latvia

**Keywords:** bed rest, central activation, muscle contractile properties, muscle function, neuromuscular junction stability, neuromuscular electrical stimulation

## Abstract

Disuse induces a disproportionate loss of muscle force compared with muscle mass, with unclear effects on voluntary muscle activation (VA) and peripheral contractility. Furthermore, the effect of neuromuscular electrical stimulation (NMES) as a disuse countermeasure remains uncertain. We investigated the effects of NMES during bed rest on neuromechanical function to improve our understanding of the mechanisms underlying disuse‐induced reductions in muscular force. Young (*n* = 16, 25 years old) and old (*n* = 16, 71 years old) adults underwent 5 days of bed rest. One leg received NMES (3 × 30 min/day), while the other served as the control (CON). Maximal isometric knee‐extensor strength (MVIC), VA and peripheral muscle contractility were assessed before and after bed rest using the interpolated twitch technique, along with biomarkers of neuromuscular junction instability (C‐terminal agrin fragment (CAF)) and muscle damage (creatine kinase (CK)). MVIC decreased in both age groups, regardless of NMES (young: CON, −21.7 Nm and NMES, −23.8 Nm; old: CON, −18.5 Nm and NMES, −16.4 Nm). VA was preserved with NMES, while decreasing in CON legs (young, −8.1%; old, −5.6%) following bed rest. Peripheral contractility (resting doublet twitch force) was reduced in CON and NMES legs in both age groups (young: CON, −4.0 Nm and NMES, −11.5 Nm; old: CON, −5.9 Nm and NMES, −10.8 Nm), with a greater decrease in NMES legs. CAF remained unchanged, whereas CK levels increased in young participants, albeit remaining within the normal range. In conclusion, a decline in neuromechanical function was observed after 5 days of bed rest in young and old adults. Although NMES appeared to preserve VA, peripheral muscle contractility was altered, resulting in reduced MVIC.

## Abstract (highlights)

- **What is the central question of this study?**
  Although disuse induces a disproportionate loss of muscle force relative to muscle mass, the effectiveness of neuromuscular electrical stimulation as a countermeasure and the mechanisms underlying disuse‐induced reductions in muscle force remain unsolved.
- **What is the main finding and its importance?**
  In young and old adults, 5 days of sustained bed rest led to reduced maximal muscle force, driven by central and peripheral impairments in neuromechanical function. The lack of strength retention with neuromuscular electrical stimulation during bed rest might be attributed to peripheral factors, because voluntary activation was preserved throughout the intervention.

## 1 INTRODUCTION

Disuse owing to bed rest represents a serious clinical concern because of the detrimental impact of mechanical unloading, resulting in muscular atrophy and impairments in mechanical muscle function (Di Girolamo et al., 2021; Mulder et al., 2015; Reidy et al., 2017; Tanner et al., 2015).

The effects of disuse on muscle mass are well documented, although several studies have noted disproportionately greater parallel impairments in mechanical muscle function irrespective of age (Hvid et al., 2010, 2014; Inns et al., 2022; Marusic et al., 2021; Monti et al., 2021; Reidy et al., 2017; Suetta et al., 2009; Tanner et al., 2015). Specifically, after 5 days of bed rest, the ratio of relative muscle atrophy to strength decline has recently been reported to be 4.2, indicating that the decline in strength is about 4‐fold greater than the atrophy response in the initial phase of disuse (∼5 days), with the ratio stabilizing at 1.9 after 1 month of sustained bed rest (Marusic et al., 2021). The disproportionate relationship is likely attributed to alterations in both central and peripheral factors, beyond muscle mass. Campbell et al. (2019) demonstrated that changes in neuromuscular function were the primary drivers of muscle strength loss during the initial stages of disuse, whereas losses in muscle mass become a more significant factor during prolonged periods of disuse (Campbell et al., 2019), although substantial myocellular atrophy (−10%) has been observed after only 4 days of immobilization (Suetta et al., 2012).

Examples of such adaptations in central factors, in terms of reduced voluntary muscle activation (VA), have been reported after 4–14 days of immobilization (Deschenes et al., 2008; Gondin et al., 2004; Hvid et al., 2018; Sarto et al., 2022; Suetta et al., 2009), accompanied by parallel changes in peripheral factors such as reduced evoked muscle twitch properties (independently of changes in voluntary activation) (Hvid et al., 2018; Suetta et al., 2009). Interestingly, disuse studies of 4–14 days duration involving both older and younger participants have reported decreases in VA assessed by superimposed twitch interpolation in old but not young participants (Deschenes et al., 2008; Hvid et al., 2018; Suetta et al., 2009). In contrast, single twitch muscle force evoked at rest, reflective of peripheral contractile properties, has been demonstrated to be reduced to a similar extent in young and old adults with short‐term (4–14 days) disuse (Hvid et al., 2018; Suetta et al., 2009), altogether indicating that the mechanisms of disuse‐induced reductions in neuromechanical muscle function might differ between young and old adults.

Throughout the lifespan, occasional periods of muscular disuse will emerge owing to injury, illness or surgery, in turn causing significant impairments in functional capacity (Coker et al., 2015; Kortebein et al., 2008; Zisberg et al., 2011). Thus, identifying effective countermeasures against disuse‐induced declines in neuromechanical muscle function seems crucial, especially for ageing individuals. Neuromuscular electrical stimulation (NMES) can be used to evoke muscle contractions in the human body (Maffiuletti et al., 2023) and offers an intervention modality that does not rely on voluntary movements or active participation (Herzig et al., 2015; Maffiuletti et al., 2013).

Although active resistance training remains the most effective approach for enhancing maximal muscle strength (Maffiuletti et al., 2023; Rahmati et al., 2021) and neuromuscular function (Aagaard, 2003; Hortobágyi et al., 2021), NMES has also been demonstrated to cause improvements in maximal muscle strength and muscle mass (Gondin, Duclay et al., 2006; Gondin, Guette et al., 2006; Rahmati et al., 2021). The observed increases in muscle strength following short‐term NMES have been suggested to include neural adaptations (Hortobágyi & Maffiuletti, 2011), exemplified by increases in VA, assessed by superimposed interpolated twitch techniques (ITTs) and evoked V‐wave recordings elicited during ongoing maximal voluntary contraction efforts following 5 weeks of NMES (Gondin, Duclay et al., 2006, 2006). In contrast, when applied during short‐term (≤7 days) disuse, NMES does not seem to mitigate the deleterious effects of disuse fully, as reductions in muscle strength have been observed despite preservation of muscle mass (Dirks et al., 2014; Reidy et al., 2017). Collectively, these observations suggest that the magnitude of disuse‐induced impairments in muscle strength might outweigh the beneficial effects of NMES in preserving muscle mass with disuse. Given the effectiveness of NMES in retaining muscle mass during short‐term disuse, the explanation for the concurrent loss in muscle strength seems to lie within the neural system.

Therefore, the aim of the present study was to examine the effects of NMES during short‐term bed rest on maximal muscle strength, VA and electrically evoked muscle twitch force (as a proxy of intrinsic muscle contractility) in young and old adults. Furthermore, we intended to compare age‐related responses to bed rest alone versus bed rest with concurrent NMES to elucidate the effect of ageing on these conditions. It was hypothesized that bed rest would cause decrements in lower‐limb muscle strength, VA and electrically evoked muscle twitch properties, whereas NMES would counteract these impairments by preserving VA and protect against disuse‐induced reductions in peripheral muscle contractility. We further hypothesized that the expected neuromechanical impairments in non‐stimulated limbs would be of greater magnitude in old compared with young adults.

## 2 MATERIALS AND METHODS

### 2.1 Ethical approval

The study received approval from the Ethical Committee of the Capital Region of Copenhagen (H‐20038614) and was registered on clinicaltrials.gov (NCT05617222). All study procedures adhered to the *Declaration of Helsinki*, and written informed consent was obtained from all study participants.

### 2.2 Participants

Potential participants, who responded to our advertisements in local newspapers and internet media, were screened for eligibility to exclude individuals with health issues, physical disabilities and/or injuries and individuals who would not be able to comply with the strict bed rest protocol. In total, 32 healthy young and old adults were recruited.

### 2.3 Experimental protocol

The experimental protocol, with 5 days of sustained bed rest with concurrent NMES, has been described in detail elsewhere (Hansen et al., 2024).

**FIGURE 1:**
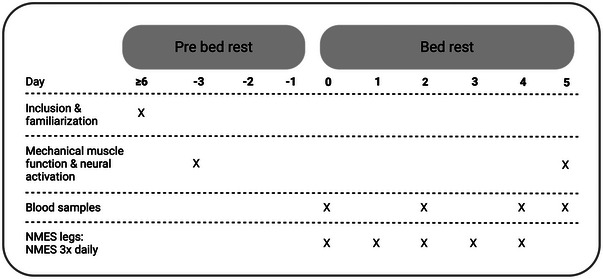
Schematic illustration of the study set‐up. In total, 32 healthy young (*n* = 16) and old (*n* = 16) females and males (50%/50%) were included and familiarized with the testing procedures. Pre‐intervention testing was conducted on day −3. On day 0, participants arrived in the Lab in the morning in the fasting state. After collection of blood samples, the bed rest was initiated. Throughout the 5 days of bed rest, NMES legs received 30 min of neuromuscular electrical stimulation three times daily, the last session being in the evening on day 4. Additional blood samples were collected on days 2 and 4, and on day 5, before participants were transported by wheelchair to the laboratory for post‐intervention testing.

Prior to the intervention period, participants were familiarized with the testing procedures. On the following visit to our Lab, participants were baseline tested (day −3) (Figure 1). Three days later, the participants returned to the Lab (day 0) in the morning, where blood samples were collected in the fasting state. Subsequently, participants were assigned to a bed in the Geriatric Ward at Bispebjerg Hospital, Copenhagen, Denmark, where they would spend the next 5 days in strict bed rest (Figure 1). Participants were asked to refrain from any weight‐bearing using their legs and to maintain as little activity as possible during the period of bed rest. The participants were allowed to be in a sitting position when needed and otherwise maintain a supine position throughout the entire bed rest period. Post‐intervention testing was conducted on day 5 (Figure 1) at the same time of day and by the same investigator as pre‐intervention testing.

Prior to commencing the bed rest, the dominant and non‐dominant legs of the participants were randomly allocated to control (bed rest only; CON) or NMES (bed rest + NMES), stratified by sex and age.

During the 5 day bed rest period, the NMES leg received three daily 30 min sessions of NMES of the quadriceps muscle, with the knee fully extended to standardize muscle length. Percutaneous muscle stimulation was applied via custom‐made compression stockings embedded with two integrated biocompatible stimulation electrodes over the proximal and distal parts of the thigh (Merhi et al., 2023). All stimulations were delivered using an electrical muscle stimulator (EMP 4 ECO+, 101060; Schwa‐medico, Germany).

Each session consisted of a 2 min warm‐up (6 Hz frequency, 300 µs pulse width) followed by a 30 min work phase (60 Hz frequency, 400 µs pulse width). The electrical stimulation was delivered using biphasic current stimulation to the muscle belly, and each stimulus was constructed as a 7 s stimulus train with a ramp‐up–ramp‐down change in stimulation intensity in a cycle: 1 s up, 5 s contraction, 1 s relaxation, followed by 10 s passive rest.

Stimulation intensity (amplitude, in mA) was increased until producing visible and palpable muscle contractions and further increased up to the highest tolerable level. An increase in stimulation intensity was allowed during each session, as tolerance increased during and between sessions.

In the morning of days 1, 2, 3 and 4, participants were asked to rate their perceived muscle soreness using a numerical rating scale ranging from 0 (no muscular soreness) to 10 (highest imaginable muscle soreness).

### 2.4 Blood samples

To explore the effects of bed rest and the impact of NMES further, we incorporated the analysis of biomarkers from fasting blood samples. Blood was sampled from an antecubital vein and collected in 9 mL tubes coated with CAT serum clot activator, in the morning of the day the bed rest would commence (day 0) and on days 2, 4 and 5 of the bed rest. Samples were stored at room temperature for between 30 min and 2 h before being centrifuged at 3172*g* for 10 min, and serum was aliquoted into Eppendorf tubes and stored at −80°C until further analysis.

C‐terminal agrin fragment (CAF) concentrations were analysed using commercially available enzyme‐linked immunosorbent assay (ELISA) kits [Human Agrin (C‐terminal fragment) ELISA Kit, A87452; antibodies.com] according to company protocol.

Extra blood samples, collected on day 0 before commencing bed rest and on day 5 before leaving the bed, were sent to the Department of Clinical Biochemistry at Bispebjerg Hospital for analysis of plasma creatine kinase (CK) levels.

### 2.5 Assessment of maximal muscle strength and neuromuscular activation

Before and after the 5 day bed rest period, maximal isometric muscle contraction force (MVIC), neuromuscular activation and evoked twitch properties were assessed for the knee extensors using a custom‐built knee dynamometer with a steel cuff connected to a strain‐gauge load cell via a rigid steel rod (Suetta et al., 2009). Participants were fastened by a seatbelt in an upright position, with a 90° flexion in hips and knees, and the external lever arm length was measured from the knee joint axis of rotation (lateral femoral condyle) to the middle of the cuff (axis of rotation of the dynamometer).

Force signals were sampled and subsequently filtered with a 20 Hz Butterworth low‐pass filter using a Bridge amplifier (National Instruments, Signal conditioning SC‐2345, Fullbridge SCC‐SG24) while data sampling was controlled from a personal computer by custom‐built software (BBH force), using a 16‐bit external analog‐to‐digital converter (NI‐USB‐6211, 16‐bit, National Instruments Corp., Austin, TX, USA). Maximal knee‐extensor torque was calculated as the product of peak strain‐gauge force and external lever arm length (Suetta et al., 2009).

Dynamometer settings were registered at the familiarization visit and used at all subsequent test sessions to ensure identical participant positioning at baseline and post bed rest.

The test protocol was performed in a fixed order: (1) determination of maximal muscle twitch response in the resting quadriceps muscle; (2) assessment of MVIC; and (3) assessment of VA during MVIC.

### 2.6 Resting muscle twitches

Each test began with an assessment of the electrical current required for evoking the maximal single twitch response in the resting muscle.

Percutaneous surface electrodes (Valutrode VL4595, 5 cm × 10 cm; Biofina A/S, Denmark) were placed over the distal (10 cm above patella) and proximal (15 cm below the anterior superior iliac spine) parts of the knee extensors (quadriceps femoris muscle) after careful preparation of the skin (Hvid et al., 2016). To ensure identical placement of the electrodes at pre‐ and post‐intervention testing, the electrode positions were marked with a permanent marker, which was maintained between test sessions (i.e., during the bed rest period).

Electrical stimulation was delivered to the resting muscles as single square‐wave pulses of 100 µs duration delivered by a direct current stimulator (model DS7; Digitimer Electronics, UK). The stimulation current was increased stepwise every 20 s until no further increase in single twitch amplitude was seen. The current was noted and delivered as a doublet stimulus during the subsequent assessment of VA (Hvid et al., 2016; Suetta et al., 2009).

### 2.7 Maximal voluntary isometric muscle strength

For the assessment of maximal muscle strength, participants performed two submaximal warm‐up trials at 50% and 80% of maximum, followed by three maximal isometric knee‐extensor contractions (MVIC) of 4–5 s duration interspaced by 45 s rest. Standardized verbal encouragement and visual online feedback of the force output on a PC screen were provided during all tests, while participants were carefully instructed to contract as fast and forceful as possible. Trials with visible countermovement (negative forces) were discarded, and another trial was performed.

### 2.8 Superimposed twitch

For evaluation of VA, participants performed two‐three additional MVIC trials, in which a supramaximal doublet twitch stimulation (100 µs pulse duration, 10 ms interpulse interval) was manually superimposed onto the MVIC at the time point of peak torque production while subsequently also elicited in the rested (potentiated) muscle ∼2 s after reaching full muscle relaxation (zero force production), the latter being used as the resting reference twitch (Hvid et al., 2016; Suetta et al., 2009).

The VA was calculated as previously described (Hvid et al., 2016; Strojnik & Komi, 1998; Suetta et al., 2009):

$$\mathrm{Activation}\left(\%\right)=100-\left\{\left[\left(D\times F_{\mathrm{Stim}}/F_{\mathrm{MVIC}}\right)/F_{\mathrm{StimRest}}\right]\times 100\right\},$$

where *D* is the difference between force at stimulation (*F*_Stim_) and the peak force evoked by doublet stimulation onto MVIC; *F*_MVIC_ is the peak voluntary force exerted before the stimulation, and *F*_StimRest_ is the evoked peak force recorded during the potentiated resting doublet. Correction of *D* (i.e., *F*_Stim_/*F*_MVIC_) was included in the equation, as the timing of the manually controlled doublet twitch did not always coincide with *F*_MVIC_ (Hvid et al., 2018; Strojnik & Komi, 1998; Suetta et al., 2009).

For each participant, the trial with the highest MVIC was chosen for further analysis using custom‐made software written in MatLab (Mathworks Inc. R2022b, MA, USA). When the analytical accuracy of the macro was in question, verification was achieved via manual curve readings.

Using a comparable set‐up, previous data from our LAb has shown excellent test–retest (i.e., repeated measurements) reliability for doublet twitch force [coefficient of variation, 2.4%; intraclass correlation (ICC_2,1_), 0.98], VA (coefficient of variation, 2.2%; ICC, 0.97) and MVIC (coefficient of variation, 3.3%; ICC, 0.99) (Hvid et al., 2018).

### 2.9 Statistical analysis

Changes pre to post bed rest in maximal muscle strength, VA and resting muscle twitch contractility, and changes in blood analyte concentrations and muscle soreness were evaluated statistically using linear mixed models (STATA/IC v.14.1; StataCorp, College Station, TX, USA) (Hvid et al., 2018, 2014).

Data were checked for normal (Gaussian) distribution using relevant STATA functions by using visual inspection of plots (‘qnorm’, ‘pnorm’ and ‘histogram’) of residuals. Furthermore, a normal distribution of blood analyte concentrations of all biomarkers and muscle soreness were assessed visually for normality using histograms and the Shapiro–Wilk test. Assurance of equal variance was conducted using the Brown–Forsythe test.

For detection of differences in response to bed rest alone (CON) and bed rest combined with electrical stimulation (NMES) within the young and old participant groups separately, participant was set as a random effect, and intervention (i.e., leg; NMES, CON) and time (pre, post) were assigned as fixed effects.

To compare pre‐to‐post changes between age groups and determine whether young and old participants responded differently to bed rest alone (CON) and bed rest combined with electrical stimulation (NMES), a mixed linear model analysis was performed on the percentage delta changes from pre to post bed rest. In this model, participant identity was set as a random effect, while leg (NMES vs. CON) and age group (young vs. old) were included as fixed effects.

When significant (*p* ≤ 0.05) main effects were detected in the mixed effects model, pairwise comparisons using *post hoc* analyses embedded in the mixed model were performed.

For the analysis of blood parameters and muscle soreness, the statistical analysis was performed using the variables time and age group (young vs. old).

Data are presented as the mean ± SD unless otherwise stated, and delta values (pre to post bed rest) are presented with 95% confidence intervals. The level of statistical significance was set at *p* ≤ 0.05 (two‐tailed).

## 3 RESULTS

In total, 32 healthy young adults [*n* = 16 (*n* = 8 females), 25.3 ± 2.1 years old, 179.1 ± 11.4 cm, 73.6 ± 14.0 kg (mean ± SD)] and old adults [*n* = 16 (*n* = 8 females), 71.2 ± 3.3 years old, 169.2 ± 9.2 cm, 73.5 ± 10.3 kg] were included in the study (Hansen et al., 2024). However, five participants (three young and two old) were excluded from the dataset owing to their inability to cooperate with the assessment of muscle activation (ITT). Thus, data from 13 young adults (8 females and 5 males; 25.0 ± 2.6 years old, 176.2 ± 10.8 cm, 70.3 ± 13.3 kg) and 14 old adults (6 females and8 males; 70.6 ± 3.0 years old, 170.5 ± 9.1 cm, 74.6 ± 10.1 kg) are reported for MVIC, VA and resting doublet twitch force.

The NMES intensity details have been reported previously (Hansen et al., 2024). Briefly, in young participants, NMES intensity increased daily by ∼10%, from 25.2 ± 4.2 mA on day 0 to 38.1 ± 9.1 mA on day 4. In older participants, the intensity increased daily by ∼9.5%, from 31.9 ± 6.3 mA on day 0 to 46.0 ± 14.1 mA on day 4. These increases were significant (*p* < 0.05), with older participants receiving higher overall stimulation intensities in comparison to young participants.

### 3.1 Maximal muscle strength

**FIGURE 2:**
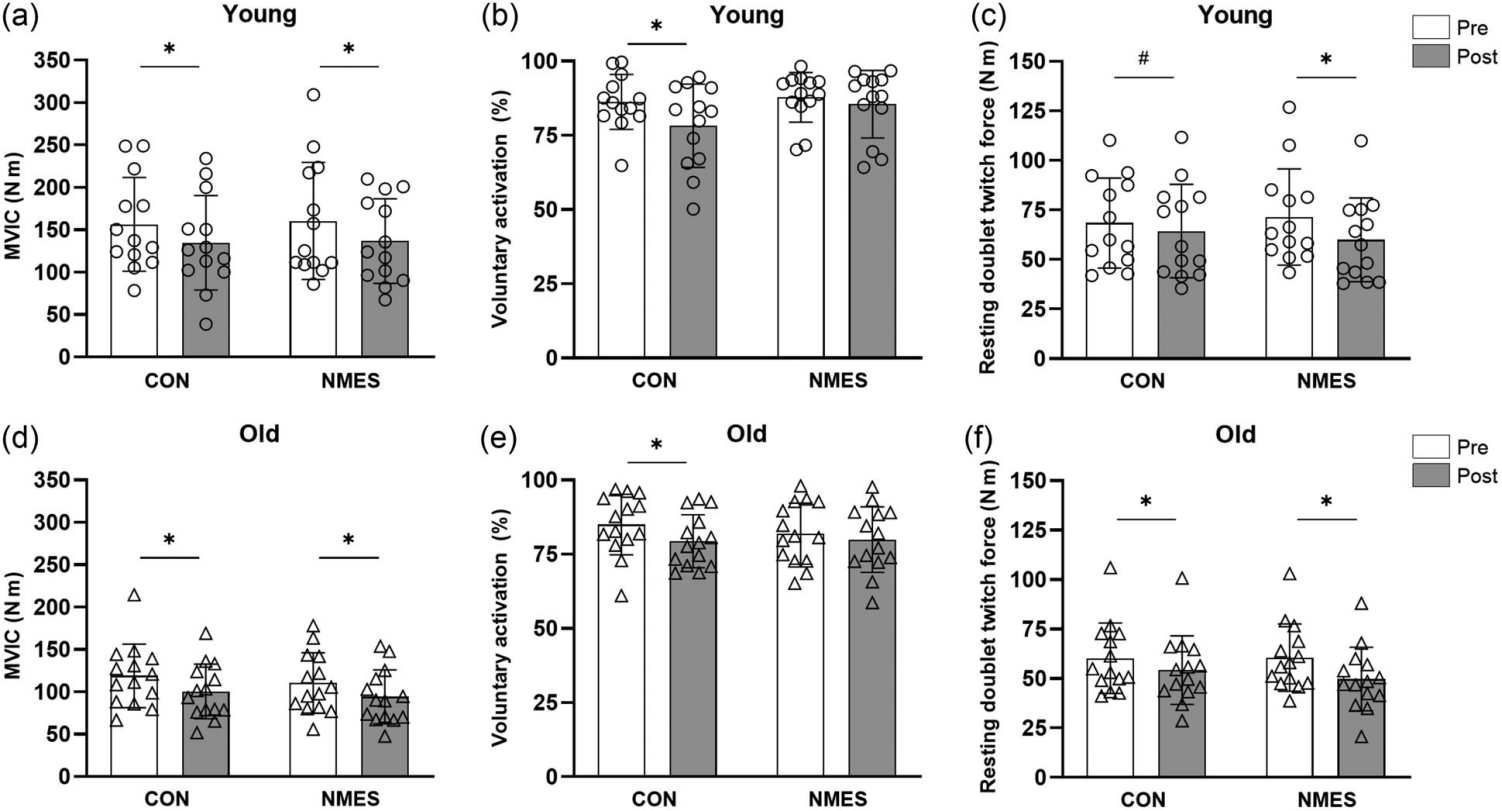
Pre and post bed rest levels of neuromechanical muscle function. (a) Maximal quadriceps muscle strength, young participants. (b) Voluntary muscle activation percentage, young participants. (c) Peripheral muscle contractility, resting doublet twitch force, young participants. (d) Maximal quadriceps muscle strength, old participants. (e) Voluntary muscle activation percentage, old participants. (f) Peripheral muscle contractility, resting doublet twitch force, old participants. Data are presented as the mean ± SD, with individual data points indicated. *n* = 13 young and *n* = 14 old. ^*^Significantly different from pre to post (*p* < 0.05). ^#^Tendency to different from pre to post (*p* = 0.087). Abbreviations: CON, control leg, bed rest only; NMES, stimulation leg, bed rest + daily neuromuscular electrical stimulation.

Following the 5 days of bed rest, maximal knee‐extensor strength (MVIC) decreased in young (main effect of time: *p* = 0.007) and old participants (main effect of time: *p* < 0.001), irrespective of NMES (Table 1; Figure 2).

**TABLE 1:** Changes in neuromechanical function pre and post 5 days of bed rest.

		Young (*n* = 13)		Old (*n* = 14)		All (*n* = 27)	
		CON	NMES	CON	NMES	CON	NMES
MVIC (Nm)	Pre	156.3 ± 55.2	160.4 ± 69.0	118.8 ± 37.4	110.3 ± 35.6	136.9 ± 49.7	134.4 ± 59.0
	Post	134.6 ± 55.8^*^	136.6 ± 49.9^*^	100.3 ± 32.2^*^	93.9 ± 31.8^*^	116.8 ± 47.6^*^	114.5 ± 46.1^*^
	Delta (Nm)	−21.7	−23.8	−18.5	—16.4	—20.0	—19.9
	[95% CI]	[–37.5; –6.0]	[–39.5; –8.0]	[–26.6; –10.5]	[–24.4; –8.3]	[–28.9; –11.2]	[–28.8; –11.1]
Activation (%)	Pre	86.3 ± 9.2	87.8 ± 8.4	85.0 ± 10.2	81.9 ± 10.3	85.6 ± 9.6	84.8 ± 9.7
	Post	78.2 ± 14.0*	85.5 ± 11.4	79.4 ± 8.9	79.9 ± 11.1	78.8 ± 11.4	82.6 ± 11.4
	Delta	—8.1	—2.3	—5.6	—2.0	—6.8	—2.1
	[95% CI]	[–13.9; –2.3]	[–8.1; 3.5]	[–9.5; –1.7]	[–5.9; 1.9]	[–10.4; –3.2]	[–5.7; 1.4]
Resting doublet	Pre	68.4 ± 22.6	71.4 ± 24.2	60.2 ± 17.8	60.6 ± 17.0	64.2 ± 20.3	65.8 ± 21.1
Twitch force (Nm)	Post	64.4 ± 23.5^a^	60.0 ± 21.1^*^	54.3 ± 17.3^*^	49.7 ± 16.1^*^	59.1 ± 20.8^*^	54.6 ± 19.0^*^
	Delta (Nm)	—4.0	—11.5^b^	—5.9	—10.8^b^	—5.0	—11.1^b^
	[95% CI]	[–8.6; 0.6]	[–16.1; –6.8]	[–9.1; –2.8]	[–14.0; –7.7]	[–7.8; –2.2]	[–13.9; –8.4]

*Note*: Values are the mean ± SD. Absolute delta values are shown with [95% confidence intervals].

Abbreviations: CON, control leg, bed rest only; NMES, stimulation leg, bed rest + neuromuscular electrical stimulation.

^a^ Tendency to different from pre (*p* = 0.087).

^b^ Significant interaction (leg × time) (*p* < 0.05).

* Significantly different (*p* < 0.05) from pre.

Analysis within each age group revealed MVIC to decrease in both CON and NMES legs following bed rest. Specifically, *post hoc* testing revealed a decrease in young (pre vs. post: 156.3 ± 55.2 vs. 134.6 ± 55.8 Nm, *p* = 0.007) and old (118.8 ± 37.4 vs. 100.3 ± 32.2 Nm, *p* < 0.001) CON legs, while decreases also were observed in young (160.4 ± 69.0 vs. 136.6 ± 49.9 Nm, *p* = 0.003) and old (110.3 ± 35.6 vs. 93.9 ± 31.8 Nm, *p* < 0.001) NMES legs (Table 1; Figure 2).

To compare the pre‐ to post‐intervention responses between age groups, we analysed the delta values of MVIC. No significant differences were observed between young and old participants. Given that no differences between the young and old participants were detected, data from both young and old NMES legs and from both young and old CON legs were pooled, respectively (NMES_all_ and CON_all_) for additional analyses of adaptive changes in MVIC. No significant intervention × time interaction was detected for the pooled data (*p* = 0.986; Table 1).

### 3.2 Voluntary muscle activation

The VA decreased in young (main effect of time: *p* = 0.006) and old (main effect of time: *p* = 0.005) participants following 5 days of bed rest, irrespective of legs (Table 1; Figure 2). *Post hoc* testing within the young and old age groups, respectively, revealed that VA was reduced in the CON legs for young (*p* = 0.006) and old (*p* = 0.005) participants, whereas no change was detected in NMES legs for either young (*p* = 0.437) or old (*p* = 0.319) participants (Table 1; Figure 2).

To determine potential differences in the pre‐ to post‐intervention changes in VA between young and old participants, we analysed the delta values (pre‐to‐post bed rest) across the two age groups. No differences were observed in delta values between young and old participants or between CON and NMES limbs. Given that no age‐related differences were detected, data from young and old participants were pooled into NMES_all_ and CON_all_ to increase statistical power. Subsequent analysis of the pooled data set (NMES_all_ and CON_all_) demonstrated a strong tendency towards an interaction effect (intervention × time; *p* = 0.070; Table 1).

### 3.3 Resting muscle twitch contractility

Peripheral muscle contractility [i.e., electrically evoked muscle twitch force (resting doublet twitch force)] decreased in old participants (main effect of time: *p* < 0.001), whereas it did not reach statistical significance in the young participants (*p* = 0.087).

In contrast, a significant interaction effect (intervention × time) was detected for both young (*p* = 0.026) and old (*p* = 0.031) participants. *Post hoc* testing revealed a significant reduction in resting doublet twitch force following 5 days of bed rest in old CON legs (*p* < 0.001), with a tendency towards reduction in young CON legs (*p* = 0.087) (Table 1; Figure 2). Moreover, resting doublet twitch force was reduced in NMES legs in both young (*p* < 0.001) and old (*p* < 0.001) participants, with greater reductions in NMES legs compared with the CON legs, in both young and old participants.

To compare the changes evoked by bed rest with or without NMES between young and old participants, the delta values (pre‐to‐post bed rest) in resting doublet twitch force were analysed, revealing an effect of intervention (i.e., leg; *p* = 0.002), which was not age specific. Given that no difference in the delta changes was observed between young and old participants, data were pooled according to leg (NMES_all_ and CON_all_), demonstrating a significant interaction effect (intervention × time; *p* = 0.004). *Post hoc* testing revealed a decrease in resting doublet twitch force in NMES_all_ (*p* < 0.001) and CON_all_ (*p* < 0.001) (Table 1).

### 3.4 Biomarker of neuromuscular junction instability

**FIGURE 3:**
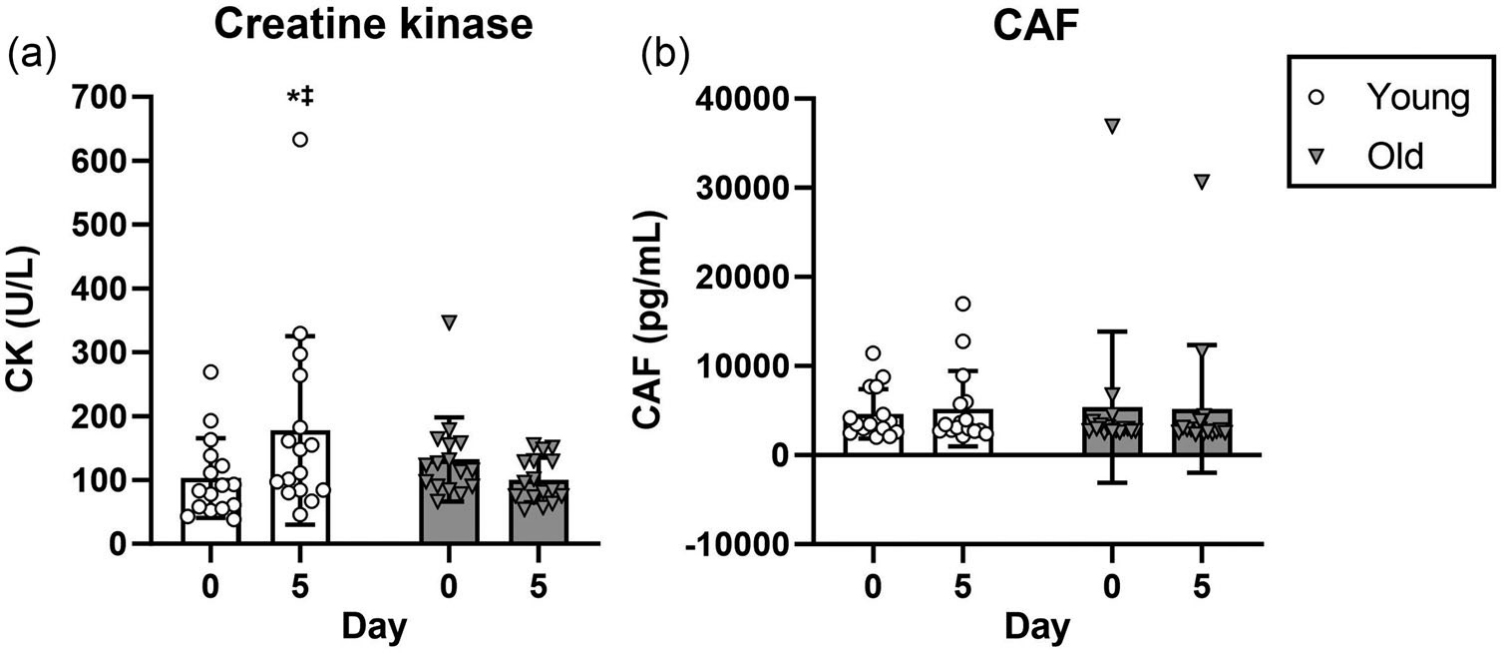
Circulating plasma creatine kinase concentration (a) and serum levels of C‐terminal agrin fragment (b) pre (day 0) and post (day 5) 5 days of bed rest with concurrent unilateral neuromuscular electrical stimulation, in young adults (circles) and old adults (triangles). Data are presented as the mean ± SD, with individual data points indicated. *n* = 16 young and *n* = 16 (old). ^*^Significantly different from pre levels. **^‡^** Significantly different from old, post bed rest. Abbreviations: CAF, C‐terminal agrin fragment; CK, creatine kinase.

Serum concentrations of CAF were measured on days 0, 2, 4 and 5. No significant change in CAF concentrations was detected at any time point (*p* > 0.05; Figure 3). Furthermore, no differences between age groups were detected during the period of bed rest.

### 3.5 Biomarkers of muscle damage and soreness

Prior to bed rest, circulating CK levels remained within normal values for young and old males and females. Following the period of bed rest, a significant interaction effect (age × time) was identified (*p* = 0.003). *Post hoc* analysis revealed that CK levels increased in young participants following the 5 day bed rest (pre, 103.1 ± 62.5 U/L; post, 177.4 ± 147.7 U/L, *p* = 0.003) while remaining unchanged in old participants (pre, 132.1 ± 66.0 U/L; post, 100.0 ± 35.0 U/L, *p* = 0.202; Figure 3). Before the bed rest, no differences were detected between young and old participants (*p* = 0.337), whereas young participants demonstrated higher CK levels post bed rest compared with old participants (*p* = 0.011) (Figure 3).

Muscle soreness in the NMES legs was rated on days 1, 2, 3 and 4 using the numerical rating scale (0–10; 0 = no pain and 10 = unbearable pain). In young participants, muscle soreness was rated as 0.8 ± 0.9, 0.8 ± 1.4, 1.1 ± 2.1 and 1.6 ± 2.1, and in the old as 0.0 ± 0.0, 0.3 ± 0.9, 0.1 ± 0.3 and 1.3 ± 2.6, on days 1, 2, 3 and 4, respectively. An overall effect of time was detected (*p* = 0.050), and *post hoc* testing revealed muscle soreness to be higher on day 4 compared with all other days (*p* < 0.005).

## 4 DISCUSSION

We investigated the effects of NMES during bed rest on neuromechanical function in young and old adults, with a focus on the disuse‐induced reductions in muscular force and the potential protection from this by means of NMES. Our main finding was that NMES was effective in preserving VA in young and old participants during 5 days of sustained bed rest. In contrast, NMES was unable to retain evoked muscle twitch forces and maximal voluntary muscle strength during the period of bed rest, irrespective of age. At the same time, no indications of altered neuromuscular junction stability or muscle damage emerged.

### 4.1 Adaptive changes in maximal muscle strength

After the 5 day bed rest period, maximal muscle strength decreased in both young adults (approximately −14%–15%) and old adults (approximately −15%), irrespective of NMES. These findings are in agreement with previous reports of declines in muscle strength following short‐term disuse, with concomitant NMES (Dirks et al., 2014; Reidy et al., 2017) and without (de Boer et al., 2007; Reidy et al., 2017; Suetta et al., 2012; Tanner et al., 2015).

Muscle thickness measurements were conducted in the present study (data not shown; Hansen et al., 2024), demonstrating that NMES effectively prevented disuse atrophy. In the CON leg, vastus lateralis thickness decreased by 5%–6% following 5 days of sustained bed rest (Hansen et al., 2024), underlining the disproportionate relationship between disuse‐induced atrophy and reduction in muscle strength (∼13%–15%), corroborating previous reports (Deschenes et al., 2002; Marusic et al., 2021; Sarto et al., 2022; Tanner et al., 2015). Compellingly, MVIC was reduced to a comparable extent in both NMES and CON legs, supporting the hypothesis that disuse‐induced reductions in muscle strength might stem mainly from alterations in the neural system.

### 4.2 Adaptive changes in voluntary activation

VA could be retained with daily sessions of NMES, while conversely decreasing in control limbs not receiving NMES during the 5 days of bed rest. The present disuse‐induced declines in VA observed in non‐stimulated control limbs align with previous observations (Hvid et al., 2018; Sarto et al., 2022; Suetta et al., 2009), although contrasting findings have also been reported (de Boer et al., 2007; Monti et al., 2021). Specifically, using the interpolated twitch technique, a 6% decline in central neural drive was demonstrated previously in young adults following 10 days of unilateral lower‐limb suspension (Sarto et al., 2022), whereas no changes were detected in young adults after 10 days of bed rest (Monti et al., 2021) or 14 days of unilateral lower‐limb suspension (de Boer et al., 2007). In contrast, declines in VA have been reported in old but not young adults after 4 days (Hvid et al., 2018) and 14 days (Suetta et al., 2009) of unilateral lower‐limb immobilization.

The disparate results reported in the literature hinder a deeper understanding of the specific neural and muscular mechanisms evoked by short‐term disuse with or without NMES. The contrasting observations may arise from methodological differences, such as variances in disuse models (including movement restriction during disuse) and limitations associated with the interpolated twitch technique (Shield & Zhou, 2004).

Using NMES as a training modality during conditions with normal weight‐bearing (i.e., free‐living conditions) has previously been shown to elicit significant increases in maximal muscle strength and VA (Gondin et al., 2005; Maffiuletti et al., 2002). Likewise, the present observations indicate that NMES might effectively counteract the negative effects of 5 day bed rest on the neural activation of myofibres during MVIC.

Previous studies have reported adaptive changes in the neuromuscular system at the spinal and/or supraspinal levels, indicated by elevated evoked V‐waves measured during maximal voluntary plantar flexion, along with reduced interpolated twitch responses (indicating increased VA) after 4–8 weeks of NMES in normal non‐immobilized conditions (Gondin et al., 2005; Gondin, Duclay et al., 2006; Maffiuletti et al., 2002). Specifically, Maffiuletti et al. (2002) speculated that the NMES‐induced enhancement in VA was driven by alterations in cortical descending drive, leading to increased motor unit (MU) recruitment and/or elevated MU firing rates. They further argued that the increase in MU recruitment, rather than changes in discharge rate, likely played a predominant role in this enhancement (Maffiuletti et al., 2002).

This hypothesis is corroborated further by evidence of enhanced VA and EMG activity observed during acute NMES of the contralateral leg, suggestive of an augmentation in both MU recruitment and the discharge rate of active MUs (Cattagni et al., 2018). Notably, declines in VA observed after 2 weeks of disuse have been suggested to result, at least in part, from decreased MU recruitment and lowered firing rates (Gondin et al., 2004). Although the precise mechanisms underlying the present retainment of VA in response to NMES were not examined, it is plausible that NMES mitigated the potential disuse‐induced decline in MU recruitment and/or MU firing rate, resulting in preserved VA in NMES legs.

### 4.3 Adaptive changes in resting muscle twitch contractility

The present study demonstrated a decrease in electrically evoked muscle twitch force (i.e., attenuated resting doublet twitch forces) in young and old adults. Interestingly, the impairment in resting doublet twitch force was larger in NMES legs (−15.6, −18.7; −17.2%), compared with CON legs (−6.8, −10.0; −8.5%) in young and old adults separately and when all data were pooled.

Although in contrast to our initial hypothesis, peripheral impairments following NMES have been reported previously (Zory et al., 2010). Zory et al. (2010) observed a decrease in peripheral muscle contractile properties after 4 weeks of NMES, which, however, returned to baseline after 4 weeks of detraining. This transient decline was described as an overreaching effect induced by NMES. In the present study, the observed decrease in resting doublet twitch force with NMES may be tied to overreaching as well, potentially through mechanisms akin to those suggested by Zory et al. (2010), involving sustained contractile excitation–contraction coupling failure. Additionally, we speculate that the present NMES protocol, with three 30 min sessions daily at maximal stimulation intensity, might have been excessive, as, unlike voluntary contractions, NMES does not allow for adjustments in MU recruitment, due to fixed parameters throughout the stimulation bout (Bickel et al., 2011; Spector et al., 2016). Hence, the myofibres might have been excessively stimulated without sufficient restitution within each session owing to the on–off duty cycle (i.e., 7 s contraction–10 s rest), and the three daily sessions, each separated by ∼3.5–4.5 h, resulting in the peripheral impairments we have observed, potentially as an overreaching effect.

Disuse‐induced alterations in excitation–contraction coupling mechanisms have been reported previously after 2 weeks of immobilization (Gondin et al., 2004) and 10 days of bed rest (Monti et al., 2021). Hence, given that disuse and NMES separately have been suggested to impact mechanisms of excitation–contraction coupling negatively, leading to peripheral impairments, it may be speculated that the present decrease in evoked muscle twitch force observed in CON and NMES legs might, at least to some degree, have been attributable to such alterations. Specifically, the more pronounced reductions in maximal resting twitch forces observed in the present study with NMES suggest a cumulative effect, whereby the negative impact of disuse and NMES may have exacerbated each other.

Collectively, these factors might have contributed to an overreaching effect, including excitation–contraction coupling impairments, as discussed above. Notably, the prospect of subsequent recovery from such overreaching remains unexplored, as post‐intervention testing was performed <24 h after the last NMES session. Given that MVIC force, VA and doublet peak torque have been reported to be suppressed ≤4 days after an acute bout of NMES (100 Hz, 400 µs; Fouré et al., 2014), the aspect of potential overreaching seems relevant for the present results. However, further research is needed to address this aspect.

### 4.4 Adaptive changes in structural biomarkers

Increased serum levels of CAF, a biomarker of muscle dysfunction related to neuromuscular junction stability (Monti et al., 2023), have previously been reported following 10 days of bed rest (Monti et al., 2021) and in response to 10 days of unilateral limp suspension (Sarto et al., 2022). In the present study, no changes in CAF concentrations were observed, which aligns with the data from Monti et al. (2021), after 5 days of bed rest in young men. This could indicate that alterations in neuromuscular junction stability do not occur within the initial phase of disuse or that potential alterations may be undetectable in systemic blood samples.

As mentioned above, it can be speculated that the peripheral impairments observed in the NMES leg may potentially involve impairments in excitation–contraction coupling. Nonetheless, it is notable that muscle soreness, albeit significantly elevated, remained <2 (on a numerical rating scale ranging from 0 to 10), which is categorized as mild symptoms (Frey‐Law et al., 2013). Additionally, circulating levels of CK, an indirect marker of muscle damage, exhibited no significant changes in the older participants but showed minor increases (+76 ± 139 U/L) in younger participants. Thus, the present study revealed no strong indications of muscle damage, underscoring the discussion above regarding other determining factors for the adaptive changes in peripheral muscle function.

### 4.5 Methodological considerations

In the present study, we used a within‐subject design to investigate the effects of bed rest with concurrent NMES in young and old female and male participants. This approach allowed for direct comparison within the same individual, effectively controlling for inter‐individual variability. However, when using a within‐subject control design (stimulated vs. non‐stimulated legs) combined with unilateral NMES, the potential for NMES‐induced cross‐education must be considered, because this phenomenon has been observed previously in contralateral homologous muscle groups (Cattagni et al., 2018; Hortobágyi & Maffiuletti, 2011; Hortobágyi et al., 1999). Notably, acute application of NMES, even at low doses (10% MVIC), has been demonstrated to increase MVIC torque and voluntary activation on the non‐stimulated contralateral side (Cattagni et al., 2018).

Given that cross‐education effects were not specifically investigated in the present study, drawing definitive conclusions on this aspect would be speculative. However, if cross‐education had occurred in the present study, the declines observed in the CON legs may have been underestimated, because the real declines could have been more pronounced. This potential underestimation should be considered when interpreting the comparison between the NMES and CON legs. Moreover, the present results may represent a more conservative estimate of the impact of NMES relative to the sustained bed rest.

Given these considerations, future research should explore the potential for cross‐education effects in similar experimental contexts and include a free‐living control group to provide a more rigorous comparative framework.

The present findings are derived from assessments of neuromechanical muscle function using the superimposed ITT both during MVIC testing and in resting conditions. This method provides a useful proxy for neural central drive by estimating how much of the maximal force capacity of the muscle is produced during voluntary contraction (Hvid et al., 2018; Taylor, 2009), while when applied at rest, it reflects peripheral muscle contractility. However, the ITT methodology has inherent limitations, as elaborated previously (de Haan et al., 2009; Shield & Zhou, 2004; Taylor, 2009), including that the output from ITT procedures is based on the mechanical output of muscle force rather than directly assessing the descending neural drive to the motoneurons (Taylor, 2009). These limitations must be considered when interpreting VA data, including the present results.

However, despite practical limitations, ITT is considered sufficiently reliable to detect changes resulting from physiological interventions (Taylor, 2009), making it applicable to the present study conditions. Nevertheless, the present results should be interpreted with these methodological constraints in mind.

In the present study, we obtained doublet twitch force to represent peripheral muscle contractility, although evoking tetanic twitch force might have served as a more ideal option. However, we refrained from this approach owing to the substantial discomfort associated with tetanic (i.e., multi‐train) electrical stimulation. Nonetheless, the present doublet twitch force recordings were deemed relevant to evaluate the pre‐ to post‐intervention changes in peripheral muscle contractility evoked by short‐term (5 day) bed rest.

### 4.6 Perspectives

Notably, although young and old participants showed similar adaptations to bed rest and concurrent NMES, old participants demonstrated lower absolute levels of maximal isometric muscle strength than their young counterparts. Clinically, this indicates that older adults might be at a greater risk of descending below a critical threshold of muscle function as a result of short‐term disuse, owing to their reduced reserve capacity. This can potentially expose older adults to a vicious circle, resulting in successive periods of disuse, consequently increasing the risk of adverse events, such as falls and fractures, ultimately leading to a reduced or complete loss of independence. Consequently, it becomes crucial to develop tailored interventions to counteract the detrimental effect of disuse, especially for the older populations.

Future studies should investigate whether an adjusted NMES protocol can preserve maximal muscle strength during short‐term disuse while addressing the preservation of reserve capacity in older adults. Additionally, future experiments should investigate the potential long‐term impact of NMES on functional performance and quality of life in this population.

## 5 CONCLUSION

In conclusion, the present study demonstrated that NMES can preserve central neural activation during 5 days of sustained bed rest in young and old adults, while evoked muscle twitch force was reduced despite NMES intervention, along with reductions in maximal muscle strength. No indications of reduced neuromuscular junction stability or muscle damage were observed.

Collectively, the present data indicate that in the absence of NMES intervention, the decrease in maximal muscle strength during short‐term disuse (bed rest) emerges as a result of both central and peripheral impairments in neuromechanical function. The lack of retainment of maximal muscle strength with disuse despite concurrent NMES intervention could be ascribed to peripheral impairments, given that VA was preserved during the intervention period. More research is warranted to explore this aspect more fully, by performing post‐intervention testing of neuromechanical function at later time points, using low‐volume NMES protocols to minimize the risk of overreaching and prevent impairments in peripheral muscle properties.

## AUTHOR CONTRIBUTIONS

Conceptualization and design: Sofie K. Hansen, Per Aagaard, Jakob Agergaard, Flemming Dela and Charlotte Suetta. Data acquisition and experimental work: Sofie K. Hansen, Pernille Hansen, Hans D. Grønbæk, Tania W. Berry, Camilla M. Olsen, Youssif Merhi and Shweta Agarwala. Data analyses and interpretation: Sofie K. Hansen, Pernille Hansen, Hans D. Grønbæk, Tania W. Berry, Per Aagaard, Lars G. Hvid, Jakob Agergaard, Flemming Dela and Charlotte Suetta. Writing—original draft: Sofie K. Hansen. Writing—revision and approval of final version: Sofie K. Hansen, Pernille Hansen, Tania W. Berry, Hans D. Grønbæk, Camilla M. Olsen, Youssif Merhi, Shweta Agarwala, Per Aagaard, Lars G. Hvid, Jakob Agergaard, Flemming Dela and Charlotte Suetta. All authors agree to be accountable for all aspects of the work in ensuring that questions related to the accuracy or integrity of any part of the work are appropriately investigated and resolved. All persons designated as authors qualify for authorship, and all those who qualify for authorship are listed.

## CONFLICT OF INTEREST

None declared.

## Data Availability

All data generated and analysed during this study are available from the corresponding author upon reasonable request. The data are not publicly available owing to privacy or ethical restrictions.
